# Racial and Ethnic Differences in Emergency Department Utilization and Diagnosis for Sports-Related Head Injuries

**DOI:** 10.3389/fneur.2019.00690

**Published:** 2019-07-02

**Authors:** Todd W. Lyons, Kelsey A. Miller, Andrew F. Miller, Rebekah Mannix

**Affiliations:** Division of Emergency Medicine, Boston Children's Hospital and Harvard Medical School, Boston, MA, United States

**Keywords:** concussion, head injury, children, emergency department, race, ethnicity, disparities

## Abstract

**Background:** Prior studies have shown racial differences in concussion awareness and outcome.

**Objective:** To assess if racial or ethnic differences exist in Emergency Department (ED) utilization and diagnosis for children with sports-related head injuries.

**Methods:** We performed a retrospective, cross-sectional analysis of ED visits from 2008 to 2017 using National Electronic Injury Surveillance System (NEISS) data. Population-weighted ED visits for children age 7–18 years with a sport-related injury were included. We compared the probability of an ED visit being for an injury to the head or diagnosed as a concussion between children of different races/ethnicities. Analyses were adjusted for age, gender, sport, year, and location where the injury occurred.

**Results:** We identified 11,529,994 population-weighted ED visits for pediatric sports-related injuries, of which 1,497,717 (13.0%) were injuries to the head and 619,714 (5.4%) received a diagnosis of concussion. Black children were significantly less likely than non-Hispanic white children to have their ED visit be for an injury to the head [Odds Ratio (OR) 0.72, 95%CI 0.65–0.79] or concussion (OR 0.58, 95%CI 0.50–0.68). Black children presenting to the ED with an injury to their head were less likely than non-Hispanic white children to be diagnosed with a concussion (OR = 0.71, 95%CI 0.59–0.85).

**Conclusions:** Racial differences exist in both ED utilization for pediatric sports-related head injuries and in the diagnosis of concussion. Further work is needed to understand these differences to ensure all brain injured athletes receive optimal care, regardless of race.

## Introduction

Sports-related injuries are one of the most common causes of head injuries and concussions in children in the United States (1, 2). Recently, concerns have been raised about the long-term effects of concussions on neurocognitive function, including the risk of chronic traumatic encephalopathy (CTE) and other neurodegenerative disorders (3–6). As a result, there have been significant efforts to increase awareness and recognition of sports-related head injuries, as well as to define protocols for concussion management and return-to-play guidelines (7, 8). Without proper recognition and diagnosis, athletes may prematurely return to play, potentially exacerbating their underlying head injury and putting themselves at increased risk of second-impact syndrome (7, 9, 10). Therefore, the identification of patients with sports-related head injuries and concussion has important implications for how young athletes, parents, and coaches approach return to play, rehabilitation, as well as the long-term risks/benefits of continued sports participation after a sports-related head injury. The Emergency Department (ED) represents a common location of initial evaluation of sports-related head injuries and concussions for children, with ED visits for young athletes increasing (1, 11).

Previous data demonstrate racial disparities in recognition of concussion symptoms and knowledge about the significance of concussion, with white athletes having higher knowledge about concussions then their African American counterparts (12). Furthermore, racial differences have been observed in the management of pediatric head injuries, with race being an important predictor of which children with head injuries undergo emergent neuroimaging (13, 14). However, it is not known whether these disparities translate into differences in patterns of ED utilization for sports-related head injuries. Nor is it known if race impacts the probability that a head-injured child will be diagnosed with a concussion. Therefore, our objectives were to: (1) assess if race/ethnicity affects the likelihood that a sports-related ED visit is for a head injury; (2) to determine if race/ethnicity impacts the likelihood that an athlete presenting to the ED with a head injury is diagnosed with a concussion.

## Materials and Methods

### Study Design

We performed a retrospective, cross-sectional analysis of ED visits from the National Electronic Injury Surveillance System (NEISS) over 10 years between 2008 and 2017. The NEISS is a division of the United States Consumer Product Safety Commission (CPSC) and reports population-weighted estimates of injuries and consumer product related injuries. The NEISS collects data from approximately 100 hospitals located throughout all geographic regions of the United States (15, 16). Emergency Department records are reviewed at the end of each day by study research coordinators and coded according to the NEISS coding manual. Clinical information including diagnostic evaluations such as laboratory testing and diagnostic imaging results are not available in NEISS. The NEISS has been utilized for multiple published reports on the epidemiology of injury (17–20). Included in the NEISS are ED visits for sports-related injuries, coded by sport of participation. This study was approved by the Institutional Review Board of Boston Children's Hospital.

### Study Population

The unit of analysis for our study was the population-weighted ED visit. We included all ED visits for children age 7–18 years with an ED visit that occurred while participating in the following sports: baseball, basketball, cheerleading, field hockey, football (American), gymnastics, ice hockey, lacrosse, rugby, soccer, softball, tennis, track and field, volleyball, and wrestling. Included in the NEISS are ED visits for injuries, as well as for medical problems such as heat-exhaustion. ED visits in our analysis include those that occurred in both organized as well as recreational sports participation, as these are not distinguished in the NEISS database.

### Study Definitions and Outcomes

We abstracted the following data elements from the NEISS database: age, sex, race, ethnicity, year of presentation, sport of participation, location where the injury occurred, body part injured, and final diagnosis. The NEISS classifies each ED visit by both body part involved and final diagnosis. We categorized injuries by the involved body region into: head, neck, torso, upper extremity, lower extremity, and other. “Other body region injuries” included heat-stroke/exhaustion, cardiac events and injuries to the ear, eye, and face. ED visits were categorized into the following final diagnoses: concussions, fractures/dislocations, sprains/strains/contusions, internal injuries, and other. “Other diagnoses” included: accidental poisonings, amputation, anoxia, avulsion and crush injuries, dental injuries, dermatitis, foreign bodies, frostbite, hematoma, hemorrhage, lacerations, nerve damage, puncture wounds, heat-related injuries, and injuries not otherwise classified. Final diagnoses were assigned by the treating ED provider and classified based on the previously defined NEISS methodology. When multiple injuries or diagnoses are present, NEISS classifies the visit according to the most severe injury (15).

NEISS classified race as White, Black/African American, Asian, American Indian/Alaska Native, Native Hawaiian/Pacific Islander, and other. In NEISS “other” includes patients who indicate more than one race or for whom none of the above categories applies. A patient's ethnicity is separately coded in NEISS as Hispanic or non-Hispanic. For our analysis, we categorized race and ethnicity into the following categories: non-Hispanic white, Hispanic, black, Asian, and other (included all patients not categorized by our aforementioned categories) (21).

The primary outcomes for our analysis were an ED visit for an injury to the head region of the body, or an ED visit for an injury diagnosed as a concussion.

### Analysis

We first compared patient characteristics between ED visits for all reasons to those for head injuries and injuries diagnosed as concussions. We describe continuous variables using medians and interquartile ranges and categorical variables using proportions and 95% confidence intervals (CI). For our primary outcome, we calculated the odds of an ED visit being for an injury to the head region or diagnosed as a concussion by race/ethnicity. For our secondary outcome, we calculated the odds of a patient with an injury to the head region being diagnosed as a concussion by race/ethnicity. We performed sub-analyses stratified by sport of participation to evaluate if sport of participation was an important confounder in the relationship between race and head injury/concussion diagnoses.

Our analyses were performed using survey-weighted logistic regression with statistical weighting provided by the NEISS (15) and results are reported as odds ratios and associated 95% CIs. Multivariate analyses evaluating the association between race/ethnicity and ED visits for injuries to the head region or diagnosis of concussion were adjusted for age, sex, year of visit, location where the injury occurred, and sport (22–24). These were determined *a priori* to be important factors in determining the types of injuries that present to an ED. Sport-specific analyses were adjusted for age, sex, year of visit, and location of injury. Due to the small number of male participants in field hockey, sex was not included in models evaluating associations between field hockey and our outcomes of interest. Secondary to the small number of female and young participants in rugby, both sex and age were not included in models evaluating associations between rugby and our outcomes of interest. In our analysis of the relationship between race and having an ED visit be for a head injury or concussion, an odds ratio of <1 represents decreased odds of the ED visit being for a head injury/concussion relative to non-Hispanic white athletes. In our analysis of the relationship between race and a diagnosis of concussion among head injured athletes, an odds ratio of <1 represents decreased odds of being diagnosed with a concussion relative to non-Hispanic white athletes.

All statistical analyses were performed using SAS software, copyright 2012 SAS Institute Inc.

## Results

We identified 360,103 unweighted ED visits for pediatric sports-related injuries. These visits correspond to 11,529,994 population-weighted ED visits, including 1,497,717 (13.0%) visits for injuries to the head region and 619,714 (5.4%) visits for injuries diagnosed as concussions (Table 1). Data on race and ethnicity were available for 8,464,746 (73.4%) ED visits. We found no association between not reporting race/ethnicity and ED visits for injuries to the head region [Odds ratio (OR): 1.05, 95%CI 0.91–1.22] or injuries diagnosed as concussions (OR: 0.97, 95%CI 0.74–1.26).

**Table 1:** Characteristics of population-weighted Emergency Department (ED) visits for pediatric sports-related injuries.

	**All ED visits *N* = 11,529,994**	**Head injury ED visits *N* = 1,497,717**	**ED visits diagnosed as concussion *N* = 619,714**
Age, median (IQR)	13.5 (11.3-15.4)	13.5 (11.2-15.3)	13.9 (11.9-15.5)
Male sex, *n* (%)	8,218,044 (71.3)	1,080,753 (72.2)	452,299 (73.0)
**Race/ethnicity**, ***n*** **(%)**			
White, non-hispanic	5,676,695 (49.2)	780,448 (52.1)	342,904 (55.3)
Hispanic	754,286 (6.5)	93,408 (6.2)	37,005 (6.0)
Black	1,779,354 (15.4)	176,553 (11.8)	64,124 (10.3)
Asian	91,256 (0.8)	14,997 (1.0)	5,735 (0.9)
Other	163,154 (1.4)	21,065 (1.4)	9,048 (1.5)
Missing	3,065,248 (26.6)	411,245 (27.5)	160,879 (26.0)
**Year**, ***n*** **(%)**			
2008	1,140,558 (9.9)	98,252 (6.6)	39,866 (6.4)
2009	1,133,525 (9.8)	121,480 (8.1)	43,045 (6.9)
2010	1,221,117 (10.6)	145,356 (9.7)	51,198 (8.3)
2011	1,203,043 (10.4)	151,872 (10.1)	59,768 (9.6)
2012	1,260,242 (10.9)	174,270 (11.6)	71,299 (11.5)
2013	1,157,985 (10.0)	167,983 (11.2)	72,485 (11.7)
2014	1,144,094 (9.9)	164,812 (11.0)	72,556 (11.7)
2015	1,124,372 (9.8)	162,507 (10.9)	69,413 (11.2)
2016	1,084,923 (9.4)	158,513 (10.6)	69,283 (11.2)
2017	1,060,134 (9.2)	152,672 (10.2)	70,800 (11.4)
**Location of injury**, ***n*** **(%)**			
Place of recreation	5,857,505 (50.8)	784,291 (52.4)	347,746 (56.1)
School	2,668,799 (23.1)	387,834 (25.9)	174,694 (28.2)
Not recorded	2,379,814 (20.6)	259,186 (17.3)	80,766 (13.0)
Home	494,912 (4.3)	50,626 (3.4)	11,791 (1.9)
Other	128,694 (1.1)	15,780 (1.1)	4,717 (0.8)
**Sport**, ***n*** **(%)**			
Baseball	935,242 (8.1)	132,139 (8.8)	34,306 (5.5)
Basketball	3,173,919 (27.5)	312,136 (20.8)	108,840 (17.6)
Cheerleading	331,063 (2.9)	50,564 (3.4)	19,597 (3.2)
Field hockey	36,940 (0.3)	5,202 (0.3)	2,210 (0.4)
Football	3,442,602 (29.9)	522,011 (34.9)	259,025 (41.8)
Gymnastics	253,909 (2.2)	16,682 (1.1)	4,911 (0.8)
Ice hockey	114,487 (1.0)	32,475 (2.2)	15,436 (2.5)
Lacrosse	151,669 (1.3)	29,909 (2.0)	12,673 (2.0)
Rugby	48,566 (0.4)	11,944 (0.8)	5,235 (0.8)
Soccer	1,531,774 (13.3)	222,474 (14.9)	93,386 (15.1)
Softball	512,320 (4.4)	63,608 (4.2)	22,183 (3.6)
Tennis	58,056 (0.5)	5,824 (0.4)	1,245 (0.2)
Track and field	222,201 (1.9)	12,955 (0.9)	3,884 (0.6)
Volleyball	362,986 (3.1)	33,147 (2.2)	13,115 (2.1)
Wrestling	354,260 (3.1)	46,646 (3.1)	23,667 (3.9)

After adjustment for age, gender, sport, year of ED presentation, and location of injury, black children were less likely to have their ED visit be for an injury to the head region or for an injury that was diagnosed as a concussion relative to non-Hispanic white children (Table 2). Similar findings were not observed for Hispanic children, Asian children or children who identified as other races. In sport-specific analyses, black children were significantly less likely than white children to have their ED visit be for a head injury if playing: basketball, football, cheerleading, soccer, or volleyball. Asian children injured playing baseball were more likely than white athletes to have their ED visits be for a head injury (Table 3).

**Table 2:** Associations between race and having an emergency department visit for a sports-related injury be for an injury to the head region or diagnosed as a concussion (compared to other body regions and diagnoses, respectively).

	**Injuries to the head region**		**Concussions**	
**Race/ethnicity**	**Unadjusted**	**Adjusted^a^**	**Unadjusted**	**Adjusted^a^**
White, non-hispanic	Ref	Ref	Ref	Ref
Hispanic	0.89 (0.60–1.30)	0.89 (0.64–1.24)	0.80 (0.46–1.36)	0.83 (0.54–1.28)
Black	0.69 (0.63–0.76)	0.72 (0.65–0.79)	0.58 (0.50–0.68)	0.58 (0.50–0.68)
Asian	1.23 (0.92–1.66)	1.25 (0.92–1.69)	1.05 (0.72–1.52)	1.08 (0.72–1.62)
Other	0.93 (0.78–1.11)	0.93 (0.78–1.11)	0.91 (0.72–1.16)	0.86 (0.68–1.10)

a *Adjusted for age, sex, location of injury, sport, and year of ED presentation*.

**Table 3:** Sports-specific adjusted associations between race and having and emergency department visit for a sports-related injury be for an injury to the brian/head region.

**Sport**	**White**	**Hispanic**	**Black**	**Asian**	**Other**
Baseball^a^	Ref	1.21 (0.84–1.75)	0.91 (0.69–1.21)	1.44 (1.02–2.03)	0.73 (0.38–1.39)
Basketball^a^	Ref	0.88 (0.50–1.19)	0.71 (0.63–0.79)	1.42 (0.90–2.24)	0.86 (0.59–1.25)
Cheerleading^a^	Ref	1.31 (0.90–1.92)	0.61 (0.46–0.81)	1.77 (0.83–3.77)	0.91 (0.41–2.03)
Field hockey^b^	Ref	0.10 (0.01–1.13)	1.16 (0.38–3.53)	^***^	0.16 (0.03–1.01)
Football^a^	Ref	1.05 (0.74–1.48)	0.71 (0.63–0.80)	1.38 (0.92–2.06)	1.10 (0.90–1.35)
Gymnastics^a^	Ref	0.93 (0.47–1.81)	0.98 (0.58–1.64)	1.42 (0.70–2.88)	0.69 (0.28–1.69)
Ice hockey^a^	Ref	1.64 (0.21–12.72)	0.96 (0.32–2.87)	1.86 (0.50–6.89)	1.92 (0.72–5.11)
Lacrosse^a^	Ref	0.55 (0.30–1.02)	1.10 (0.65–1.86)	1.90 (0.73–4.91)	0.74 (0.33–1.66)
Rugby^c^	Ref	0.75 (0.27–2.07)	0.60 (0.28–1.30)	0.31 (0.07–1.44)	2.32 (0.89–6.0)
Softball^a^	Ref	1.44 (0.90–2.29)	0.79 (0.57–1.10)	0.89 (0.46–1.74)	1.10 (0.47–2.56)
Soccer^a^	Ref	0.67 (0.54–0.84)	0.72 (0.57–0.92)	0.92 (0.60–1.39)	0.70 (0.57–0.87)
Tennis^a^	Ref	0.54 (0.21–1.44)	1.33 (0.64–2.79)	1.39 (0.35–5.58)	0.90 (0.16–5.12)
Track and field^a^	Ref	0.56 (0.30–1.07)	0.81 (0.53–1.24)	0.84 (0.43–1.62)	1.14 (0.47–2.81)
Volleyball^a^	Ref	0.88 (0.51–1.53)	0.67 (0.45–0.98)	1.23 (0.63–2.41)	0.74 (0.36–1.51)
Wrestling^a^	Ref	1.09 (0.77–1.55)	0.79 (0.56–1.10)	0.79 (0.52–1.19)	0.94 (0.38–2.23)

a *Adjusted for age, sex, location of injury, and year of ED presentation*.

a *Adjusted for age, location of injury, and year of ED presentation*.

a *Adjusted for location of injury and year of ED presentation*.

*** *Unable to calculate due to small number of weighted ED visits*.

Among the sub-set of children presenting to the ED with an injury to their head region, black children were less likely than non-Hispanic white children to be diagnosed with a concussion (Table 4). In contrast, we found no significant difference in rates of concussion diagnoses between head-injured, non-Hispanic white children and Hispanic children, Asian children or children who identified as other races. In adjusted analyses, other factors associated with receiving a diagnosis of concussion among pediatric patients presenting to the ED with an injury to the head region included: age (OR 1.08, 95%CI 1.07–1.10 for each year over age 7), female gender (OR 1.18, 95%CI 1.09–1.29), injuries that occurred at places of recreation (OR 2.06, 95%CI 1.58–2.67) or school (OR 2.08, 95%CI 1.58–2.67) compared to those injuries occurring at home, and year of presentation (OR 1.08, 95%CI 1.04–1.10 for each year after 2008). Among children presenting to the ED with a head injury, the greatest differences in the diagnosis of concussion were found in basketball, cheerleading, football, gymnastics, and soccer (Table 5).

**Table 4:** Association between race and having an ED visit with an injury to the head region be diagnosed as a concussion.

**Race/ethnicity**	**Unadjusted**	**Adjusted^a^**
White, non-hispanic	Ref	Ref
Hispanic	0.84 (0.61–1.14)	0.84 (0.65–1.10)
Black	0.73 (0.62–0.86)	0.71 (0.59–0.85)
Asian	0.79 (0.62–1.02)	0.85 (0.65–1.12)
Other	0.96 (0.73–1.27)	0.86 (0.64–1.17)

a *Adjusted for age, sex, sport, location of injury, and year of ED presentation*.

**Table 5:** Sport-specific adjusted associations between race and the diagnosis of concussion in athletes presenting to the emergency department with an injury to the head region.

	**White**	**Hispanic**	**Black**	**Asian**	**Other**
Baseball^a^	Ref	0.98 (0.60–1.61)	0.72 (0.50–1.04)	1.03 (0.55–1.93)	1.06 (0.44–2.56)
Basketball^a^	Ref	0.61 (0.40–0.94)	0.71 (0.57–0.90)	1.12 (0.63–2.01)	1.00 (0.61–1.63)
Cheerleading^a^	Ref	1.28 (0.70–2.35)	0.56 (0.32–0.99)	0.87 (0.32–2.35)	1.44 (0.47–4.47)
Field hockey^b^	Ref	^***^	^***^	^***^	^***^
Football^a^	Ref	0.88 (0.63–1.24)	0.68 (0.54–0.85)	0.65 (0.47–0.88)	0.77 (0.48–1.24)
Gymnastics^a^	Ref	2.39 (0.50–11.3)	0.22 (0.07–0.68)	0.80 (0.11–5.92)	21.57 (1.93–241.35)
Ice hockey^a^	Ref	1.07 (0.08–13.89)	0.52 (0.14–1.98)	0.56 (0.09–3.59)	18.50 (1.88–181.74)
Lacrosse^a^	Ref	0.60 (0.12–2.99)	0.71 (0.30–1.68)	0.07 (0.02–0.25)	0.44 (0.13–1.52)
Rugby^c^	Ref	0.56 (0.10–3.06)	0.80 (0.23–2.75)	1.57 (0.05–46.52)	2.71 (0.43–16.92)
Softball^a^	Ref	1.05 (0.60–1.84)	1.39 (0.77–2.51)	0.29 (0.05–1.81)	0.87 (0.12–6.61)
Soccer^a^	Ref	0.78 (0.63–0.97)	0.76 (0.47–1.20)	0.91 (0.64–1.30)	0.71 (0.36–1.38)
Tennis^a^	Ref	^***^	^***^	^***^	^***^
Track and field^a^	Ref	0.71 (0.05–9.78)	0.62 (0.26–1.47)	0.24 (0.02–2.95)	0.45 (0.12–1.62)
Volleyball^a^	Ref	1.20 (0.57–2.53)	0.57 (0.28–1.17)	0.81 (0.30–2.23)	1.47 (0.54–4.04)
Wrestling^a^	Ref	1.18 (0.66–2.09)	1.16 (0.51–2.66)	0.70 (0.23–2.13)	0.28 (0.05–1.73)

a *Adjusted for age, sex, location of injury, and year of ED presentation*.

b *Adjusted for age, location of injury, and year of ED presentation*.

c *Adjusted for location of injury and year of ED presentation*.

*** *Unable to calculate due to small number of weighted ED visits*.

**Figure 1:**
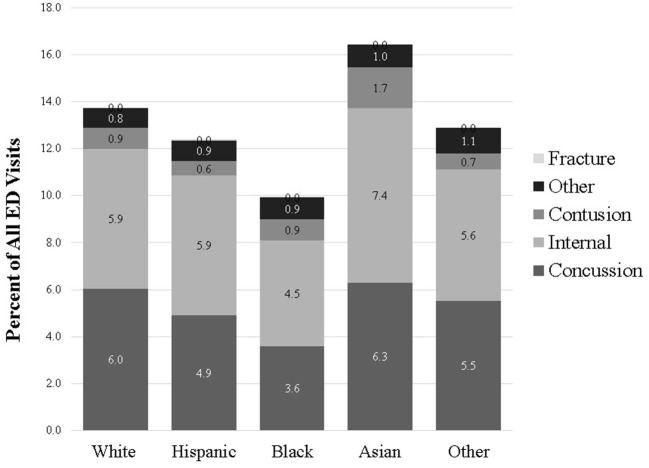
Percent of ED visits for sports-related injuries attributed to head injuries and sub-divided by final diagnosis.

Differences in the percent of children presenting with a head region injury and those diagnosed as a concussion are summarized in Figure 1. The difference in diagnoses was driven by a higher percentage of black children with head injuries receiving a diagnosis of contusion, internal injury, or “other injury.” We found no significant differences in the rates of diagnosis of fracture among children with injuries to the head region.

## Discussion

Among ED visits for sports-related injuries, black children were nearly 30% less likely than non-Hispanic white children to have their ED visit be for an injury to the head region and over 40% less likely to have their ED visit be for the diagnosis of a concussion. Among children presenting to the ED with an injury to the head region, black children were more than 30% less likely to be diagnosed with a concussion. In addition, we found increasing rates of ED visits for sports-related head injuries and concussion over time. These findings are consistent with prior data and may represent increased awareness and diagnosis rather than an absolute increase in the actual number of concussions. The results of our analysis raise important questions about how race affects the decision to seek ED level care for a sports-related head injury, as well as how race impacts the likelihood that a head-injured athlete receives a diagnosis of concussion.

Prior research has demonstrated racial differences in concussion knowledge and recognition of concussion symptoms (12). Specifically, white athletes in a prior study demonstrated increased concussion knowledge and more frequently recognized the signs and symptoms of concussion. In addition, racial disparities in the use of neuroimaging in evaluation of head injuries in the ED have been reported (13, 14). In these two studies, white children were more likely to have emergent neuroimaging (MRI or CT) performed than black children. Finally, racial differences have been reported in the site of initial care for brain-injured children (25), with black children more likely to have their point of entry into the health care system for a concussion be in the ED. Our study is consistent with these prior data, demonstrating racial differences in the evaluation and management of head injured children. However, while prior data have shown the ED is a common site of initial care for brain-injured black children, our data demonstrate significant racial differences in care utilization and diagnosis in the ED setting, with black children less likely to have an ED visit be for a head injury or concussion. Taken together, these findings suggest the magnitude of underutilization and underdiagnosis may be even greater than we observed. Finally, our study is the first to our knowledge to demonstrate how race affected ED utilization and diagnosis for athletes with head injuries.

We found that among athletes with an ED visit for a sports-related injury, black children were less likely to have that visit be for a head injury or diagnosed as a concussion. While our study demonstrates racial differences in ED utilization, our findings could represent either overutilization of the ED for head-injured non-Hispanic white athletes or underutilization by black athletes. The observed racial differences in ED utilization could be due to a variety of factors including differences in the recognition of signs and symptoms of head injuries, differences in level of concern about head injuries, access to certified athletic trainers, differences in where patients seek initial care for head injuries, and differences in referral patterns to the ED for head injuries (12, 25, 26).

We found that black children were 30% less likely than their non-Hispanic white counterparts to be diagnosed with a concussion when presenting with an injury to the head region. Head injured black children were more likely than their white counterparts to receive a general diagnosis of head contusion or internal injury. It is possible that many of these children receiving these diagnoses did in fact have a concussion. These findings raise important questions about potential racial bias in the evaluation and diagnosis of head-injured athletes (27). It is not clear if our finding represents under-diagnosis of concussion in young, black athletes, or over- diagnosis in non-Hispanic white athletes. Importantly, the differences were most pronounced for football, soccer, basketball, and cheerleading. These results are significant as these sports have been associated with high rates of head trauma, including repetitive head trauma (24). Because most clinically important traumatic brain injuries can be ruled out without the need for emergent neuroimaging (28), most ED visits for children with sports-related concussions focus on education regarding cognitive rehabilitation and return to play guidelines (7). With increasing research and guidelines around concussion management and return to play, diagnostic precision is becoming increasingly important. Racial differences in the clinical diagnosis of concussion could result in disparities in the post-injury care of children with sports-related head injuries and concussions. Specifically, broad diagnoses like head “contusion” or “internal injury” may not signal to athletes, their parents, trainers, and coaches that a concussion has occurred. In the absence of a diagnosis of concussion, these athletes may return to play prematurely, and risk repeat head injury before the brain has had adequate time to recover. Conversely, if over diagnosed, children with concussion could be inappropriately held out of play. Therefore, clinicians must ensure that all athletes receive the appropriate diagnosis and return to play instructions, regardless of race.

The findings of our study must be interpreted in the context of its limitations. First, a significant proportion of children in our study did not report race. However, we found no significant association between not reporting race and having an ED visit be for a head injury or concussion. Second, we only evaluated care in the ED, meaning our findings may not be generalizable to other care settings. Prior work has suggested that only evaluating ED care for concussion may significantly underestimate the incidence of pediatric head trauma (25). Third, we had limited clinical data on children in our analysis and could not assess what clinical factors contributed to the diagnosis of concussion. However, it is unlikely that patterns of head injuries vary by race, after adjustment for age, gender, sport, year, and location where the injury occurred. Fourth, we are unable to assess if sports-related head injuries occurred in the context of organized or recreational sport participation. Last, some of the observed differences in the diagnosis of concussion among athletes with injury to the head region may reflect race-related differences in case-mix. However, the observed difference in our study was driven by an increased proportion of head-injured black children being diagnosed with “internal injuries,” “contusion,” or “other.” While it is possible these differences may represent an increased incidence of intracranial hemorrhages in head-injured black children, these diagnoses in sports are rare and unlikely to vary to by race or ethnicity. Therefore, the observed disparities likely represent differences in how the diagnosis of concussion is given to head-injured athletes in the absence of other more significant clinical diagnoses.

In conclusion, we identified race-based differences in ED utilization for injuries to the head region and injuries diagnosed as concussions. We also found race-based differences in the diagnosis of concussion among young athletes who presented to the ED with injuries to their head region. Further work is needed to understand factors leading to these observed differences to help ensure that all head-injured athletes receive optimal care, regardless of race.

## Ethics Statement

This study was approved by the Institutional Review Board of our hospital with a waiver of informed consent, given the anonymous nature of the dataset.

## Author Contributions

TL conceptualized and deigned the study, performed the data analyses, drafted the initial manuscript, and reviewed and revised the manuscript. KM and AM aided in the conceptualization and design of the study, reviewed data analyses, and reviewed and revised the manuscript. RM conceptualization and designed the study, supervised data analyses, and reviewed and revised the manuscript. All authors approved the final manuscript as submitted and agree to be accountable for all aspects of the work.

### Conflict of Interest Statement

The authors declare that the research was conducted in the absence of any commercial or financial relationships that could be construed as a potential conflict of interest.
